# The significance of microbiome in personalized medicine

**DOI:** 10.1186/s40169-019-0232-y

**Published:** 2019-05-13

**Authors:** Ava Behrouzi, Amir Hossein Nafari, Seyed Davar Siadat

**Affiliations:** 1 Department of Mycobacteriology and Pulmonary Research, Pasteur Institute of Iran, Tehran, Iran; 2 Microbiology Research Center (MRC), Pasteur Institute of Iran, Tehran, Iran

## Abstract

Considering the important role of microbiome, many of current investigations have focused on its beneficial aspects. Although, research explores new dimensions of the impact of microbiome and examines the differences in patients and healthy individuals for identifying biomarker patterns, but limited information is available, and investigation in this field seems to be of great value. On the other hand, new therapeutic approaches, called personalized medicine, have opened a new window in medical science, and the association between microbiome and personalized medicine seems to be one of the most interesting aspects of the subsequent research, and has a pivotal perspective on the treatment of diseases such as cancer. Accordingly, given the novelty of the relationship between these two axes, there are very few studies in this regard. The presence of specific strains may have the ability to modulate cancer progression and therapeutics; this increases the likelihood of precision medicine in relation to microbiota, in terms of treatment and prognosis, and therefore, microbiota is a next generation medicine and may develop a novel therapeutic action in this field.

## Background

Microbial communities, including bacteria, archaea, fungi, etc., are known as microbiota or microflora, and the genes encoded by them are called microbiome. A healthy microbiome has a series of joint characteristics that can be distinguished from non-healthy individuals, so understanding the microbiome differential properties may contribute in detection and identification of the disease-associated microbiome. The microbiome of healthy people is very diverse with a high number of beneficial microbes that can withstand the changes, occurring during each period of physiological stress; while the disease-associated microbiota is less diverse; the number of beneficial bacteria is lower and leads to the disease in the presence of inflammation [41, 65]. However, the main problem that the researchers are facing is to understand the potential characteristics of microbiome diversity, among individuals [34, 53]. Traditional methods such as cultivation have provided very little information in this regard [20], but today, the approaches such as NGS have been able to introduce an acceptable understanding of this population and their combinations, and identified the archaea, bacteria and viruses in the body [40, 60, 73]. Disturbance in microbial ecology may be associated with many diseases, such as diabetes, inflammatory bowel disease and so on; human microbiome can be used as a primary diagnostic biomarker, and researchers today focus on its therapeutic role [50]. As we know, the microbiota of each human body organ is unique, and its effects on inflammation and cancer are also distinct in each organ, as well as understanding the changes in interpersonal microbiome, and the frequency of microbial population in different positions in organs, leading to information potentially related to the development of diseases such as cancer [27]. These differences may be responsible for the occurrence of cancer in a particular organ, for example, the susceptibility to colorectal cancer is due to the presence of higher microbial density, compared to the small intestine [6, 51]. The microbiome is responsible for various clinical outcomes, and the drug response of individuals can be due to these differences; not all patients show the same response to anticancer therapies. Therefore, given the consideration of each person’s genetic information, and the improvement of drug responses [12], the personalized therapeutics can play a prominent role in the health care program, especially in relation to cancer. Studies have shown that the gut microbiome can also be effective in treating cancer through the regulation of host inflammatory responses [35]; microarray techniques can be helpful in this regard, since they can simultaneously evaluate more than hundreds of cancer-related genes. Modern personalized therapeutic is integrated with each individual genetic structure and disease history before the disease begins, and this is unlike the traditional personalized therapeutic. Each tumor is a specific set of genetic patterns, so that understanding genetic alterations and gene expression profiles in cancer cells can lead to effective therapies. The National Institutes of Health (NIH) has provided a lot of information on the importance of cancer genomics in personalized treatment, and not only genomics, but also proteomics play an important role in personalized treatment [46]. Inflammation, metabolism, and genotoxicity are key mechanisms, in which microbiota can modulate carcinogenesis and can therefore be used to develop anticancer therapies [63]. Today, new therapeutic approaches, called personalized medicine, have opened a new window in medical science, and the link between microbiome and personalized medicine seems to be one of the most interesting aspects of future research and is considered as an important perspective on the treatment of diseases like cancer.

## Main text

### The role of microbiome metabolites in the development of disease (Fig. 1)

With the advent of sophisticated diseases such as cancer, the association between environmental, microbiome and cancer effects can be very complicated. Changes in cell metabolism and inflammation are a sign of cancer [30]. Even if host-microbiome reactions to cancer are not considered as an essential event, the presence of microbial compounds in some cancers, such as colorectal cancer (CRC), can be indirectly important. In vitro studies have reported a signaling process between bacterial quorum sensing peptides (QSPs) and cancer cells. Bacillus-derived QSPs are synthesized when the bacteria are under stress conditions and have the ability to induce invasive tumor cells in a process called Epithelial mesenchymal like (EMT-Like) (involved in CRC metastasis) [80]. The QSPs participate in both metastatic and angiogenesis behaviors under these conditions [69, 80]. In other types of cancers, microbial activity can reduce the effectiveness of chemotherapy [77] or affect tumor development [35]. Lifestyle and diet are also the ones that play a major role in determining the microbiome. In addition, the production of various metabolites by gut microbiota is effective in cancer-promoting and cancer-protecting induction; however, different determinants are still not fully understood [8].

**Fig. 1:**
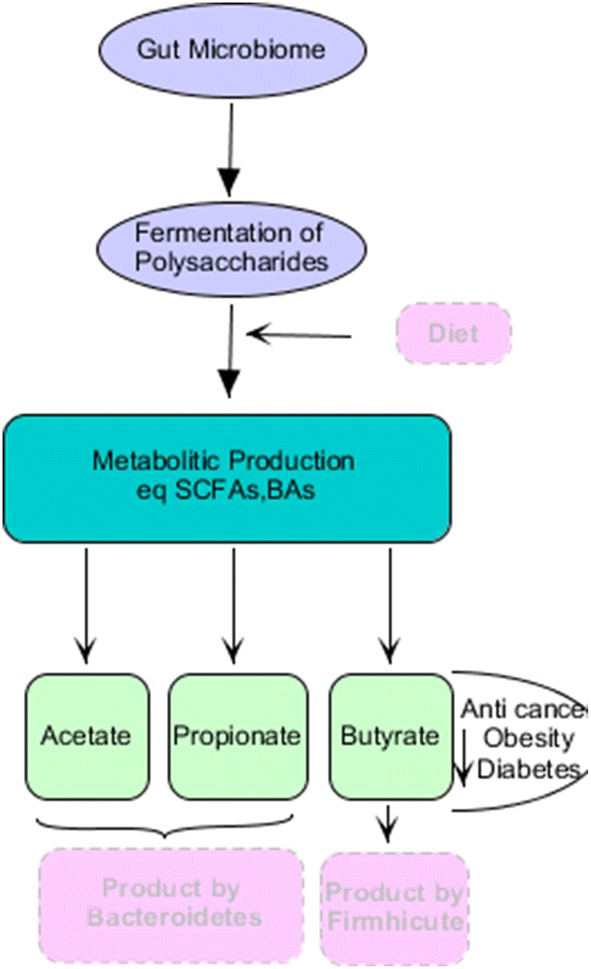
Metabolites production by microbiota and role of its on health

Microbiome-derived metabolites have the potential to contribute to cancer development, and this has been recognized [47]. Clearly, the diet is a great source of these metabolites; for example, high-fat and high-protein diets are a feature of the modern Western diet [2, 33], which is one of the risk factors for the occurrence of cancer [5, 22]. On the other hand, the bile acid (BA) is used as a signaling molecule associated with metabolic homeostasis [1]. Specific enzymes convert BA to SBA [54] that can act as a carcinogen [3]. In vitro studies have shown that the exposure of an hour to SBA compounds such as deoxycholic acid (DCA) and lithocholic acid (LCA), leads to extensive damage to DNA that has a dose-dependent behavior [4]. Studies have shown that the African-American population showed more incidence and more deaths than the Native American population relative to CRC. The microbiome combination of these two groups (African–American and Native American) was studied, and the African-American group was abundant in *Bacteroides* species, while the native group was abundant in *Prevotella* species [54]. Additionally, the encoded genes for SBA and fecal SBA in the first group had higher levels, whereas short chain fatty acids were higher in Native American, and therefore studies reported [14] that despite the same genetic history, phenotypic and developmental differences of a specific disease are possible, and these differences are mainly due to various diets and microbiome combinations. The consumption of fiber-rich foods induces saccharolytic fermentation, due to different species of gut microbes that produce short chain fatty acids and specifically acetate, propionate and butyrate [32]. For example, *Bacteroidetes* have high levels of acetate and propionate, while *Firmhicute* bacteria produce high amounts of butyrate. Some anti-cancer activity is associated with the butyrate. For instance, the butyrate can induce S-phase ablation in colorectal adenocarcinoma cells and result in growth inhibition by inducing apoptosis and expression of cell regulators such as P21 and cyclin B1 [31]. Interestingly, the butyrate effects are in cell-dependent manner; the butyrate in normal cells induces the proliferation as a source of energy, while the butyrate in cell lines inhibits the proliferation and triggers the apoptosis [13].

### The relationship between inflammation, cancer, and microbiome (Figs. 2, 3)

Chronic inflammation and inflammatory factors such as reactive oxygen and nitrogen species, cytokines and chemokines can contribute to the growth and metastasis. The microbes in relation to cancer, activating NFκB signaling, are within tumor microenvironments. The NFκB is activated in tumors with high prevalence of *Fusobacterium nucleatum (F. nucleatum)*, which is found to be abundant in colorectal cancer [23]. The NFκB is the regulator of inflammatory responses, also activator of the survival-triggering genes within cancer cells, and inflammatory-inducing genes within the microenvironment [18]. FadA is an adhesin in *F. nucleatum*. In vitro studies have shown that FadA, by binding to TIGIT inhibitory receptors in NKC, and inhibiting its cytotoxic activity, is helpful in invading immune system in tumor cells [29]. In addition to the innate immune system, microorganisms will also affect the acquired immune system; for example, as soon as a specific bacterium is exposed to the CD4 T cell, it is possible to produce cytokines that induce tumor progression [21]. For instance, IL-23 is one of these cytokines that is produced by tumor-associated myeloid cells, in response to microbial products such as flagellin, which promotes the growth and development of tumor cells and develops tumor IL-17 responses [28].

**Fig. 2:**
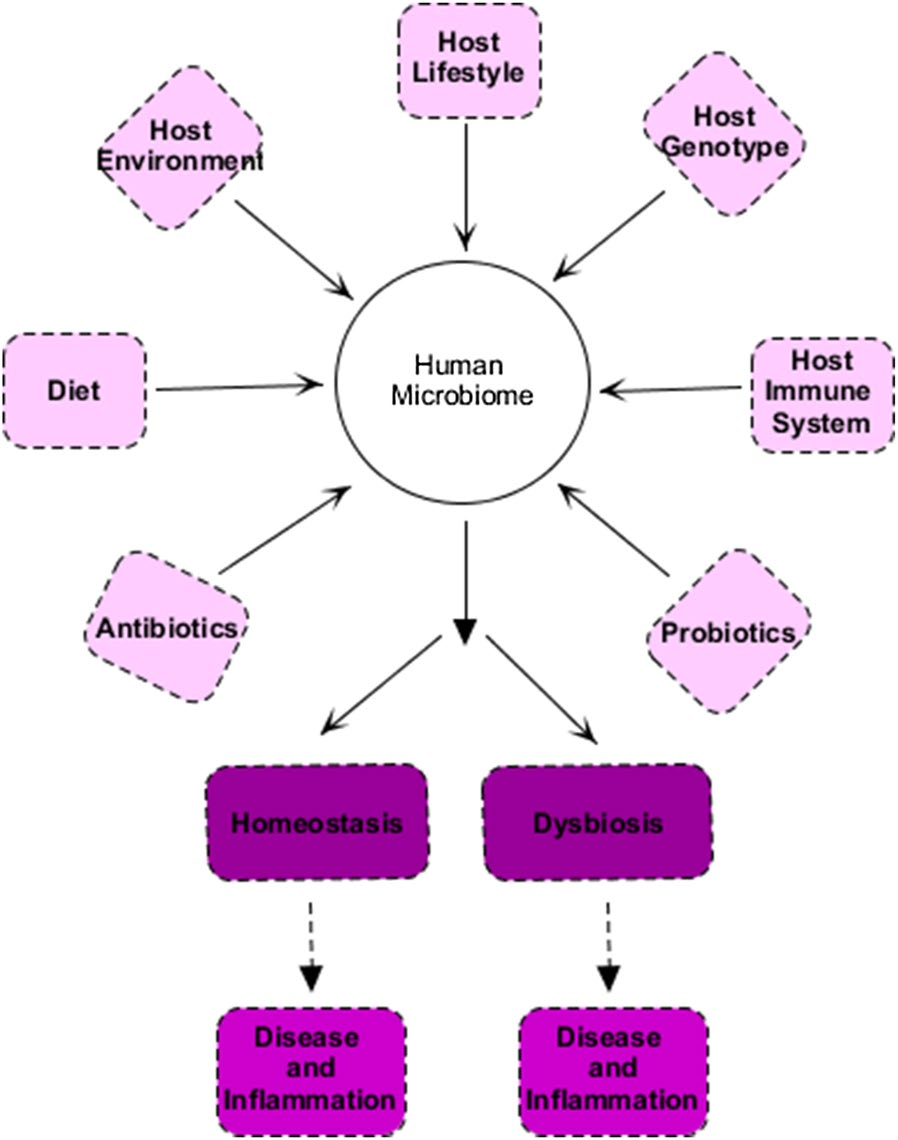
Diet, environment factors and host influence on microbiota and effect of microbiota on homeostasis and dysbiosis

**Fig. 3:**
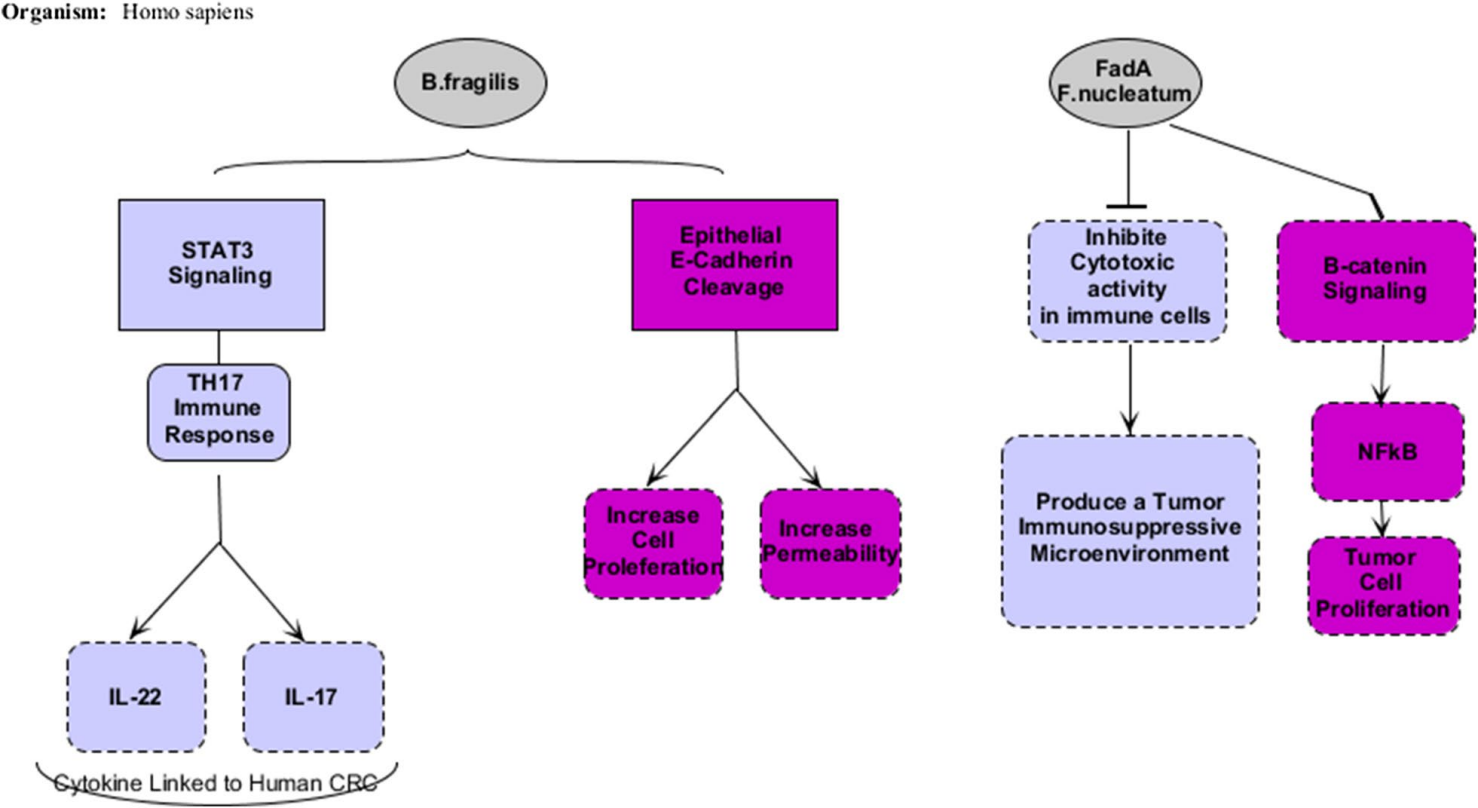
Effect of *B. fragilis* and *F. nucleatum* on inflammation and colorectal cancer

Enterotoxigenic *Bacteroides fragilis* leads to inflammation in humans and induces colitis, and strongly induces colonic tumor in multiple intestinal neoplasia of mice in vivo. This toxin induces STAT3 signaling via Th17 responses that results in the production of IL-17 and IL-22, and other cytokines linked to human colorectal cancer by activating the STAT3 pathway [36]. On the other hand, the inflammation can be associated with other malignancies and is a risk factor for cancer development; for example, obesity can be a producer of overrepresentation of bacterial species capable of producing pro-carcinogenic metabolites such as SBA. Dysbiosis in obese subjects, changes the intestinal epithelium, causing more permeability to microbial production, [47] which can activate immune cells in the lamina propria and after circulation reach the liver, and lead to the production of pro-inflammatory cytokines such as TNF and IL-6 [19]. Finally, studies have shown that barrier deterioration due to microbial products has been associated with colorectal tumorigenesis [28].

### The role of microbiome in precision diagnosis and personalized treatment

Various evidence suggests that dysregulation of microbiota-host interaction is correlated with different diseases such as IBD, diabetes, cirrhosis and colorectal cancer [45]. Recently, studies have been conducted concerning the reactions between bacteria and cancer treatment drugs [15, 35, 75], and the findings suggest that interactions of the bacteria mediated with the immune system, are necessary for drug efficacy, although little information is available on the effects of human microbiome combinations, and treatment outcomes in cancer patients [37]. Many studies [26, 48, 58] have shown that the patients in accordance with gut microbiome combinations have the potential to respond to or not respond to immunotherapy, and this can be considered in the evaluation of drug interactions. Moreover, the emergence of the role of gut microbiome as a biomarker for disease phenotype, prognosis and response to treatment, is well described in relation to the alteration of microbial population structure in various diseases [79, 78]. Discussions have revealed that the gut microbiome is associated with surgery in CD subjects along with increased mucosa-associated *F. prausnitzii* in the recurrent disease [25]. Despite many studies in relation to microbiome in IBD, there is no agreement between outcomes, because it is due to geographical differences and the use of antibiotics, diet, and other effective factors that affect the gut microbiome. Therefore, further studies on mucosal bacteria needed in relation to inflammatory diseases such as IBD. In addition, microbiome signatures are associated with many other related gastrointestinal diseases. For example, *F. nucleatum* is used as a diagnostic marker via FadA adhesin in colorectal cancer [59], or *Clostridium difficile* (*C. difficile)* infection is associated with reduced microbial diversity and low production of secondary bile acids [7, 52]. In addition, recently two studies have identified microbiome signatures in the *C. difficile* infection that can predict the disease [42, 64]. A study found remarkable results in this regard, showing that these patients had 90% clinical improvement after receiving fecal microbiota transplantation (FMT) from stool samples of healthy people [37]. Another example in this regard, was the observation of the spread of *Proteobacteria* in patients with celiac disease, which has gastrointestinal symptoms, compared to those with the same illness that exhibited extra-intestinal symptoms [76]. Many studies have been interested in describing the gut microbiota signature in the systematic disorders such as rheumatoid arthritis, and confirm that *Prevotella* is seen in these patients [55, 62]. In another study, these individuals were abundant in *Collinsella, Eggrthella* and *Faecalibacterium* [11].

Many microorganisms are associated with treatment responses. For example, it is noteworthy that patients responding to anti-PD1 therapy had a high incidence of *Faecalibacterium*, while patients who did not respond to treatment showed a high incidence of *Bacteroidale*. Studies suggested that microbial populations are a source of bacterial immune synergy to respond to anti-PD1 treatment. People with metastatic melanoma who showed a better response to treatment, had a high prevalence of *Bifidobacterium longum*. The presence of these species in the tumor-bearing rat intestine showed improved treatment for anti-PD-L1 [67]. On the other hand, two species of *Ruminococcus obeum* and *Roseburia intestinalis* were observed in people who did not respond to treatment. Routly observed that exposure to antibiotics over the course of cancer therapy can be linked to anti-PD1 treatment responses, and in fact confirms that the destruction of the microbial network and the loss of specific bacteria can interfere with the efficacy of immunity. Comparison of fecal microbiota in those, responding to treatment showed a relative increase in *Akkermansia muciniphila,* compared to those who did not respond, which also an indication of an optimal outcome for anti-PD1 therapy. The microbiota of the patients, responding to the treatment contained immunoregulatory bacteria, such as *Akkermansia*, *Faecalibacterium* and *Bifidobacterium*, which had a better performance over anti-PD1. In another study [26, 58], it was observed that mice receiving FMT from patients, who responded to treatment, experienced further recovery response to anti-PD1 treatment than the mice receiving FMT from those without treatment response. It was found that this improved response was associated with the frequency of *Faecalibacterium* in the rat stool. Later in complementary studies [48], it was found that the tumors of mice receiving FMT derived from subjects, responding to the treatment, showed a high CD8 T cell level, compared to the other group. On the other hand, Routly [26] reported that the presence of *A. muciniphila* in mice, receiving FMT derived from subjects who did not respond to treatment, resulted in an improved antitumor activity of immune cells.

Other interesting observations exhibited that the strains of *Akkermansia, Faecalibacterium* and *Bifidobacterium* are associated with anti-inflammatory responses, which is an immune system arm that prevents over-response activation, and leads to the formation and maintenance of homeostasis [10]. For instance, the relative decline of *A. muciniphila* in the intestine is associated with many diseases, such as IBD, type II diabetes and other diseases [10]. Similarly, *F. prausnitzii* downregulates intestinal inflammation related to the production of specific metabolites, such as butyrate, salicylic acid derived from host cells or bacteria in the intestines and peripheral blood [49].

All of these studies suggest that the precision medicine strategy, including gut microbiota, can have therapeutic potential. Finally, all these results suggest that efforts are being made to create synthetic microbial communities for the treatment of various diseases such as IBD and CDI. The gut microbiota has the ability to modulate the individual health, through many immune and non-immune cell types such as RNA, DNA, membrane compounds, etc. via the production of a network of metabolites. The interesting point is that in addition to having sporadic microbiota, in the case of patients who respond to treatment, one can assume that better synergy with treatment can be observed in intestinal bacteria in the event of a translocation to the secondary lymphoid organs that create a specific anti-tumor immune response.

## Discussion

The Human Microbiome Project with the mission to supply required resources and expertise to characterize the human microbiome and examine its role in health being was launched by National Institute of Health in 2007. HMP acts as a road map to discover the role microorganisms play in human health, disease, nutrition, and immunity in different parts of the body. It examines the microbes in five different areas of the body: nose, mouth, skin, vagina, and colon. It should be noted that according to the research most communities of the microbes are distinct from each other (e.g. the microbes on the skin are distinct from those in mouth, intestine, and vagina). The microbes do not also appear in mixture, and all major groups, phyla, of the bacteria that may colonize the human body, do not exist in everybody site. Two major strategies so far have been used to analyze microbial communities through NGS: shotgun metagenomics and 16S rDNA sequencing. Shotgun metagenomics is an integral part of sequencing of bacterial DNA isolated from the whole microbial community [72]. 16SrDNA sequencing relies on amplification of the polymerase chain reaction (PCR) in a specific region of the 16S gene [43]. It is assumed that 16S sequencing is a robust, well characterized method that provides adequate information about microbial communities’ composition, starting from a relatively small number of sequences per samples (*200 thousands). However, one of the major limitation of the method is assignment of the taxa based on the sequence of only a single region of the bacterial genome [56]. Shotgun metagenomics, on the other hand, requires a more complex downstream data analysis and higher coverage (10–30 million of reads). Nevertheless, shotgun metagenomics through collecting sequence information about broad genomic regions allows a more accurate definition at the species level and consequently yields a detailed description of bacterial community [66].

Human microbiome can be used to detect biomarker and present research intends to examine its therapeutic role [50]. Given that microbiome is a biomarker of diseased state, examining microbiomics and metagenomics is necessary to find out the processes. Present biomarker will be the future theranostics which could outline the suggested way of diagnostic therapy for the patients and test the new probable medication methods to find the best treatment based on the screening results.

Nevertheless, there are yet many challenges which should be addressed; for example, are we manufacturing antimicrobial drug resistant flora; how the microbiome modifies drugs, what are the side effects and how they can be minimized? [57] Gut microbiome as a tool regarding targeted non-invasive biomarkers has been established by compelling studies for certain diseases or cancers. Microbial metabolites (for example branched chain amino acids) can serve as microbial biomarkers regarding metabolic disorders like prediabetes and type 2 diabetes to prevent or mitigate the disease [74]. More than the well-defined associations of any alterations in the structure of microbial community among different kinds of disease states, gut microbiome recently has been used as a biomarker for disease prognosis, phenotype, and response to treatment [39]. In addition, Inflammatory bowel disease is one of the best examined conditions related to dysbiosis, where microbiome has served as an important marker for response to treatment and disease phenotype. An association also has been reported between gut microbiome signatures and surgical outcomes in CD, where an increase in *F. prausnitzii* in the ileal mucosa has been associated with a decrease in disease recurrence at 6 months [79, 78]. Although some studies have highlighted the changes in the microbiome in IBD, but there is no consensus in this regard. Accordingly, to overcome the effect of antibiotic use, disease subtype, and other factors affecting the gut microbiome [25], to have large cohorts from different geographic locations is a necessity. Diet in general, and consumption of dietary fiber in particular, seems to affect gut diversity, ecology, and function substantially [71]. The research shows a connection between host health and dietary MAC (Microbiota-Accessible Carbohydrates). Gut microbiota composition is affected by induced alterations in dietary fiber and microbiota composition. Nevertheless, there is large inter-individual variations [16, 68]. According to Kovatcheva-Datchary et al. improved glucose metabolism after dietary fiber supplementation leads to an increase in abundance of *Prevotella* in gut microbiota [44]. Research also shows that dietary fibers, through modulating the gut microbiota, can prevent high-fat diet induced obesity [45]. Recent studies regarding the impact of protein intake on the gut microbiota suggest that high-protein diets lead to an increase in detrimental metabolites in feces [61]. It is also reported that consumption of omega-3 fatty acids due to modulation of the gut microbiota can reduce chronic inflammation and body weight gain [38]. For example, the composition of gut microbiota in early-life stressed animals changed due to EPA/DHA. According to Degnan et al. cobalamin and related factors shape the composition of human gut microbial communities and their functions [17]. Emulsifiers as a dietary compounds alter gut barrier dysfunction and gut microbiota and have negative impacts on metabolism [9]. In addition, recent studies have identified two microbiome signatures that can be predictor of disease outcomes and allow therapeutic stratification [64]. The patients affected with celiac disease with gastrointestinal symptoms experienced an expansion of proteobacteria in the setting of dysbiotic microbiota in compare with those with extra-intestinal manifestations of celiac disease [76]. In addition to diseases within gastrointestinal tract, gut microbiome signatures is reported in systemic disorders such as rheumatoid arthritis. Expansion of intestinal *Prevotella copri* is also reported in new onset rheumatoid arthritis (RA) [62]. Enrichment of *Faecalibacterium, Eggerthella* and *Collinsella* in patients with RA and a strong correlation between *Collinsella* and high levels of asparagine and alpha-aminoadipic acid, as well as production of experimental arthritis and alpha-aminoadipic cytokine IL-17A are reported in another recent study [11].

Above mentioned examples, among the others, provide experimental evidence to prove the role of microbiome in human disease and future implications of microbiome based biomarkers for diagnostic and therapeutic purposes. Although much research has been done to identify biomarkers, but validating these signatures in large multicenter cohorts and identifying their causative role needs more combination of in vitro and in vivo models [39].

## Conclusion

The development of diagnostic tests, using biomarkers for use in primary diagnosis is one of the key aspects of precision medicine [70]. CRC is one of the cases on which the studies have been conducted, so that researchers evaluated the potential of fecal microbiota for the early detection of CRC, and applied it as a screening tool among various clinical groups of healthy people, and those with adenoma and carcinoma [81]. These limited studies have confirmed the role of microbiome in human diseases, and that the microbiome population may be used as a diagnostic and a therapeutic biomarker in the near future. Although these studies are preliminary, and there is definitely a need for in vitro and in vivo studies with more confirmatory tests for each disease, in order to achieve a suitable microbiome signature.

Further studies are needed to understand how bacteria affect the immune system and tumor microenvironments, and on the other hand, the association between microbial populations and antitumor therapy response is complicated. In fact, selective reduction and bacterial taxa by means such as exposure to antibiotics or other stressors, may result in reduced immunotherapy responses. Additionally, the presence of specific microorganisms on other sites may lead to interference with treatment [24]. For example, *E. coli,* by metabolizing and deactivating the active form of the drug, reduces the effects of chemotherapy, which can have a negative interaction with tumor responses [24], so the presence of specific strains may have the ability to modulate cancer progression and therapeutics. This increases the likelihood of precision medicine in relation to microbiota, in terms of treatment and prognosis, and therefore, microbiota is a next generation medicine and may develop a novel therapeutic role in this field.

## Abbreviations

NGS: next generation sequencing
NIH: National Institute of Health
CRC: colorectal cancer
QSPs: quroum sensing peptides
EMT-Like: epithelial mesenchymal like
BA: bile acid
DCA: deoxycholic acid
LCA: lithocholic acid
NFkB: nuclear factor kB
STAT3: Signal Transducer and Activator of Transcription 3
IBD: inflammatory bowel disease
FTM: fecal microbiota transplanation
